# Default and Executive Network Coupling Supports Creative Idea Production

**DOI:** 10.1038/srep10964

**Published:** 2015-06-17

**Authors:** Roger E. Beaty, Mathias Benedek, Scott Barry Kaufman, Paul J. Silvia

**Affiliations:** 1 Department of Psychology, University of North Carolina at Greensboro, USA; 2 Department of Psychology, University of Graz, Austria; 3 The Imagination Institute, University of Pennsylvania, USA

## Abstract

The role of attention in creative cognition remains controversial. Neuroimaging studies have reported activation of brain regions linked to both cognitive control and spontaneous imaginative processes, raising questions about how these regions interact to support creative thought. Using functional magnetic resonance imaging (fMRI), we explored this question by examining dynamic interactions between brain regions during a divergent thinking task. Multivariate pattern analysis revealed a distributed network associated with divergent thinking, including several core hubs of the default (posterior cingulate) and executive (dorsolateral prefrontal cortex) networks. The resting-state network affiliation of these regions was confirmed using data from an independent sample of participants. Graph theory analysis assessed global efficiency of the divergent thinking network, and network efficiency was found to increase as a function of individual differences in divergent thinking ability. Moreover, temporal connectivity analysis revealed increased coupling between default and salience network regions (bilateral insula) at the beginning of the task, followed by increased coupling between default and executive network regions at later stages. Such dynamic coupling suggests that divergent thinking involves cooperation between brain networks linked to cognitive control and spontaneous thought, which may reflect focused internal attention and the top-down control of spontaneous cognition during creative idea production.

Neuroscience has made substantial progress in demystifying how the brain generates novel and useful ideas. Despite progress in the field, however, several fundamental questions remain. One central question concerns the role of attention—whether creative thought involves more or less cognitive control. Past research provides seemingly contradictory evidence, reporting activation of brain regions associated with both cognitive control (e.g., dorsolateral prefrontal cortex) and spontaneous imaginative processes (e.g., the precuneus; 1). Moreover, these regions correspond to the core hubs of large-scale networks that typically act in opposition^1^. It therefore remains unclear whether their activation reflects isolated contributions or increased cooperation among regions. We explored this question by examining whole-brain functional connectivity during a divergent thinking task. A functional connectivity approach can reveal the extent to which distributed brain regions interact to support complex cognitive processes, which may shed further insight on the role of attention in creative cognition.

## Large-Scale Networks and Creative Cognition

Research in cognitive neuroscience has increasingly focused on examining large-scale functional networks^2^,^3^. Functional networks consist of spatially distributed brain regions that show a correlated pattern of activity at rest and during cognitive tasks^4^. One of the most widely studied networks is the default mode network (DMN), a set of midline and inferior parietal regions that activate in the absence of most external task demands^5^,^6^. The landmark discovery of the DMN has led to an explosion of interest in its role in attention and cognition (for reviews, see 7,8,9). The DMN is associated with cognitive processes that require internally-directed or self-generated thought, such as mind-wandering^10^, future thinking^11^, perspective taking^12^, and mental simulation^13^. The apparent overlap between these processes and those hypothesized to support imagination has fueled speculation that the DMN may be important for creativity^14^,^15^,^16^,^17^,^18^.

Recent theorizing on the role of the DMN in creativity has received support from neuroimaging studies linking individual default regions to performance on creative thinking tasks. For example, the precuneus—a core hub of the DMN^19^—has been implicated in both structural^20^,^21^,^22^,^23^ and functional^24^,^25^,^26^ imaging studies of divergent thinking. Moreover, activation of the inferior parietal lobule (IPL), another core hub of the DMN^27^, has also been reported in several neuroimaging studies of creativity^28^,^29^,^30^,^31^. For example, Benedek and colleagues^29^. found that generating new ideas during a divergent thinking task (responses participants identified as novel during functional imaging) was related to increased involvement of the left IPL, providing further support for a role of default mode regions in creative cognition.

Although the DMN and spontaneous thought appear to be important for creativity, past research also points to a role of brain regions associated with cognitive control. Supporting evidence comes from neuroimaging studies reporting activation within the lateral prefrontal cortex, a core hub of the executive control network (ECN; 3, 33). The ECN is engaged during cognitive tasks that require externally-directed attention, such as working memory^32^, relational integration^33^, response inhibition^34^, and task-set switching^35^. Regions of the ECN have been implicated in several creative thought processes, including divergent thinking^36^, artistic drawing^37^, and musical improvisation^38^. Together, such findings suggest that creativity also taps brain networks linked to the top-down control of attention and cognition.

The controversy surrounding the role of attention in creativity is also evident in behavioral research on the cognitive basis of creative thought. Although early theories emphasized unconscious and associative processes in creativity (e.g., 39), a growing body of recent evidence points to a role of cognitive control mechanisms, such as working memory capacity^40^,^41^, fluid intelligence^42^,^43^, verbal fluency^44^, and pre-potent response inhibition^45^. Such executive functions are hypothesized to support creative thought by providing the attention control needed to manage complex search processes and inhibit salient but irrelevant conceptual knowledge^45^,^46^,^47^,^48^.

## The Present Research

A growing body of research suggests that creative cognition recruits brain regions associated both cognitive control and spontaneous imaginative processes. Such work commonly implicates regions within large-scale networks, including the ECN and the DMN. Despite their apparent cooperation, evidence from resting-state and task-based research suggests that the DMN and ECN tend to act in opposition—activation of one network typically corresponds to suppression of the other^1^. This antagonistic relationship is thought to reflect opposing modes of attention, with ECN activity indicating focused external attention and DMN activity indicating spontaneous internal attention^2^.

We believe these findings may be an artifact of the measurement methods employed in cognitive neuroscience, most of which use paradigms that require focused external attention. Indeed, when attention is externally directed, such as viewing a visual stimulus, the dorsal attention network and executive networks are coupled, and both networks are anticorrelated with activity of the default mode network. Emerging evidence, however, suggests that the executive and default networks actually cooperate whenever it is necessary to perform a task that requires *extended evaluation of internal information*^8^. Under these contexts—including autobiographical future planning, positive constructive daydreaming, keeping track of social information, and evaluating creative ideas—the dorsal attention network and executive networks become decoupled and the executive network couples with the default mode network^17^,^37^,^49^,^50^,^51^,^52^. Recent research is also beginning to reveal the importance of executive and default network interactions for the healthy development of cognitive control^53^,^54^,^55^, self-regulation^56^,^57^, emotion regulation^58^,^59^, and memory suppression^60^.

To further understand the dynamic interplay between executive and default networks, we examined the time-course of brain network connectivity during performance on a creative thinking task. Participants completed an alternate-uses divergent thinking task and a control task during functional magnetic resonance imaging (fMRI). Multivariate pattern analysis was used to assess whole-brain connectivity associated with divergent thinking. Seed-based and temporal connectivity analyses explored further connections between regions identified in the whole-brain analysis. The present study thus sought to identify a whole-brain network associated with divergent thinking and to explore other potential connections between regions identified in the whole-brain analysis.

## Method

### Participants

The original sample consisted of 28 young adults from the University of North Carolina at Greensboro (UNCG). Participants received course credit or cash payment for their involvement in the study. Three participants were excluded for excessive head movement (>3 mm), resulting in a final sample of 25 (13 females; mean age: 21.04 years, age range: 18-30). All participants were right-handed with normal or corrected-to-normal vision and no reported history of CNS-affecting drugs or neurological disease. All participants provided written informed consent. The study was performed in accordance with the guidelines and regulations of UNCG’s Institutional Review Board, who approved the study methods.

### Procedure

Participants completed two tasks in the scanner: an alternate uses divergent thinking task and an object characteristics task. The alternate uses task required participants to generate creative uses for everyday objects (e.g., a brick); the object characteristics task, our control task, required participants to generate typical properties of everyday objects. These two tasks provide an optimal contrast for isolating brain activity related to the creative manipulation of objects during divergent thinking while controlling for activity related to the mental visualization of objects (see also^30^,^61^). Participants received thorough training on both tasks and completed several practice trials prior to scanning. Prior to the fMRI experiment, they also competed a timed divergent thinking task—alternate uses for a brick (2 minutes)—on a computer running MediaLab v.2010.3. Responses were subsequently coded for creative quality by three trained raters using the subjective scoring method.

The task paradigm consisted of a jittered fixation cross (4-6 s), a cue indicating the upcoming condition (“create” or “object”; 3 s), an idea generation period presenting an object in text (e.g., “umbrella”; 12 s), and a response period requiring a button press to indicate whether an idea was successfully generated (1 = *yes*, 2 = *no*; 3 s). The purpose of the response period was to ensure compliance and maintain active engagement with the task; all trials were included in the subsequent analysis. A total of 46 trials were administered in an event-related design. For each participant, experimental stimuli were randomly assigned to either condition (alternate uses or object characteristics). Participants were encouraged to continue to generate ideas until the end of the idea generation period.

### MRI Data Acquisition and Preprocessing

Participants completed the tasks in a single fMRI run. Whole-brain imaging was performed on a 3T Siemens Magnetom MRI system (Siemens Medical Systems, Erlangen, Germany) using a 16-channel head coil. BOLD-sensitive T2*-weighted functional images were acquired using a single shot gradient-echo EPI pulse sequence (TR = 2000 ms, TE = 30 ms, flip angle = 78°, 32 axial slices, 3.5 × 3.5 × 4.0 mm, distance factor 0%, FoV = 192 × 192 mm, interleaved slice ordering) and corrected online for head motion. The first two volumes were discarded to allow for T1 equilibration effects.

Visual stimuli were presented using e-Prime and viewed through a mirror attached to the head coil. Following functional imaging, a high resolution T1 scan was acquired for anatomic normalization. Imaging data were slice-time corrected and realigned using the Statistical Parametric Mapping (SPM) 8 package (Wellcome Institute of Cognitive Neurology, London). Functional volumes were coregistered and resliced to a voxel size of 2mm^3^, normalized to the MNI template brain (Montreal Neurological Institute), and smoothed with an 8 mm^3^ isotropic Gaussian kernel.

We assessed task-related functional connectivity using the CONN toolbox (http://www.nitrc.org/projects/conn; 63) in MATLAB. For each participant, CONN implemented CompCor, a method for identifying principal components associated with segmented white matter (WM) and cerebrospinal fluid (CSF; 62). These components were entered as confounds along with realignment parameters in a first-level analysis. Because CompCor accounts for the effects of subject movement, the global BOLD signal was not regressed.

### Analytic Approach

Functional connectivity analysis was conducted in four steps. First, to identify brain regions showing significantly greater functional connectivity during divergent thinking, we analyzed whole-brain connectivity with multivariate pattern analysis (MVPA; 63). Next, to determine the putative resting-state network affiliation of select regions identified in the MVPA, we conducted resting-state functional connectivity analysis using an independent sample of age-matched participants. Task-related functional connectivity analyses were then conducted with these ROIs to further explore connectivity associated with divergent thinking. Finally, graph theory methods were used to compute global efficiency of the whole-brain network of ROIs to explore whether network efficiency was modulated by individual differences in divergent thinking ability (i.e., creativity ratings of responses generated during the alternate uses task completed outside of the scanner).

### Multivariate Pattern Analysis

MVPA assesses the entire multivariate pattern of pairwise connections between all voxels in the brain^63^. First-level voxel-to-voxel covariance matrices were computed for each participant and for both tasks, permitting second-level analyses that tested for differences in whole-brain connectivity between conditions by means of a statistical F-test. In contrast to standard univariate analysis, which considers the effects of each voxel cluster separately, MVPA accounts for multivariate dependencies in the data. Thus, second-level statistical analysis yields a multivariate pattern of voxel clusters showing connectivity differences between the two task conditions (i.e., divergent thinking vs. object characteristics). But because MVPA is an omnibus test, post-hoc analyses are needed to determine specific connectivity patterns in the data^63^. We therefore extracted regions of interest (ROI; 10 mm spheres) based on peak activation clusters from the whole-brain analysis to explore further connections between these regions during the task.

### Resting-state Functional Connectivity Analysis

We conducted resting-state functional connectivity analysis with select ROIs using an independent sample of age-matched participants (*n* = 42). Past research suggests that subtle differences in ROI placement within a given brain region can affect the corresponding resting-state networks^64^. This approach therefore allowed us to determine the putative resting-state network affiliation of the ROIs. Structural and functional imaging data were acquired using the same scanning parameters described above (see MRI Data Acquisition and Preprocessing). For resting-state functional imaging, participants were asked to relax with their eyes closed for five minutes. Following functional imaging, a high-resolution T1 scan was acquired for anatomic normalization. Preprocessing steps also followed the same procedure as above, with the exception of a conventional band-pass filter applied to the resting-state time series (i.e., 0.008-0.09; 63).

### Seed-to-Voxel and ROI-to-ROI Analyses

We then conducted a series of seed-to-voxel and ROI-to-ROI analyses with the task-based data to assess pairwise correlations between the ROIs. For the seed-based analyses, we explored connectivity between select ROIs and all other voxels in the brain. For the ROI-to-ROI analysis, we explored dynamic changes in functional connectivity between ROIs across the task duration. The two task conditions (divergent thinking and object characteristics) were divided into six 2 s intervals, corresponding to the repetition time (TR) of 2 s and total task durations of 12 s. We then computed task contrasts for each of the 2 s task intervals (*p* < .05 FDR corrected). Due to the temporal lag in the BOLD signal, the first time window was not analyzed, resulting in five temporal windows for analysis (i.e., TRs 2-6).

### Graph Theory Analysis

We explored whether activity of the whole-brain network of ROIs was modulated by individual differences in divergent thinking ability using graph theory methods. For each participant, the CONN toolbox computed global network efficiency—a graph theory measure that is increasingly used to assess the integrative capacity of complex systems^65^. We focused on global efficiency as it has been shown to be one of the most robust measures of brain network integrity^66^. Global efficiency reflects effective information transfer or “small-worldness”^65^ within a network of nodes (i.e., ROIs) and edges (i.e., correlations or “paths” between nodes). It is mathematically expressed as the inverse of the average shortest path length in a graph *G* to all other nodes in the graph. For our purposes, global efficiency provided a marker of information flow within a brain network associated with divergent thinking.

Composite creativity scores were computed for each participant by averaging the subjective ratings of the three raters for the divergent thinking task completed outside of the scanner (i.e., alternate uses for a brick). Inter-rater reliability for the three raters’ scores was good (Cronbach’s alpha = .87). The global efficiency and divergent thinking composite scores were standardized by z-score transformation. Finally, we computed the correlation between global network efficiency and composite creativity scores. We hypothesized that network efficiency would be positively correlated with individual differences in divergent thinking ability.

For all second-level analyses, T-tests on Fisher’s Z-transformed correlations were used to test for differences in functional connectivity between tasks. Results are reported when significant at a voxelwise threshold of level of *p* < .001 uncorrected. Seed-to-voxel analyses are reported at a cluster-level threshold of *p* < .05 familywise error (FWE) corrected; ROI-to-ROI analyses are reported when significant at a threshold of *p* < .05 false discovery rate (FDR) corrected^63^.

## Results

### Multivariate Pattern Analysis

**Figure 1:**
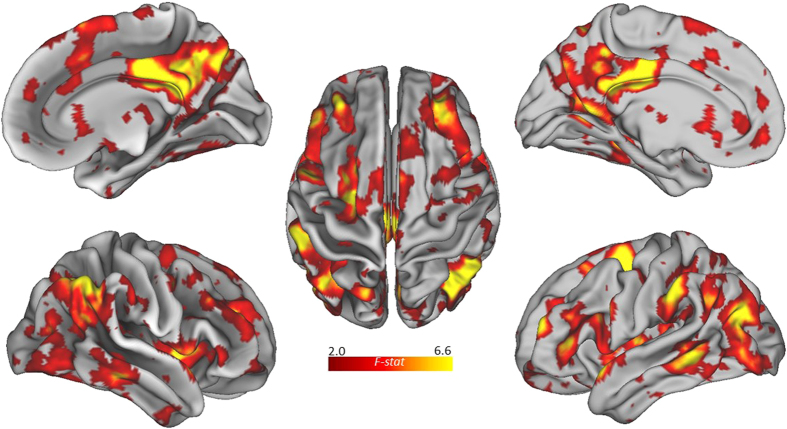
Multivariate pattern analysis for the whole-brain task contrast (alternate uses > object characteristics).

The MVPA task contrast (alternate uses > object characteristics) revealed a distributed network of voxel clusters associated with divergent thinking (see Table 1 and Fig. 1). The networkconsisted of several frontal, temporal, and parietal regions, including regions within the default network: the left precuneus, right PCC, and bilateral IPL. The network also included the right DLPFC, a core region of the ECN, as well as the right ACC and bilateral insula, core regions of the salience network^2^. In addition, the network included several significant clusters within the temporal lobes (e.g., bilateral middle temporal gyri; MTG), regions associated with semantic and episodic memory retrieval. Taken together, the whole-brain MVPA identified a distributed network of brain regions associated with divergent thinking, including several core regions of the default, executive, and salience networks.

### Resting-state Functional Connectivity Analysis

We then explored resting-state functional connectivity with select regions of the DMN (left precuneus and right PCC) and ECN (right DLPFC) associated with divergent thinking in the MVPA. This analysis was conducted using data from an independent sample of participants (*n* = 42). In line with previous resting-state research, we expected that the PCC and precuneus seeds would show positive correlation with other default network regions (e.g., MPFC) and negative correlation with executive network regions (e.g., DLPFC); likewise, we expected that the DLPFC seed would show positive correlation with other executive network regions and negative correlation with default regions.

**Figure 2:**
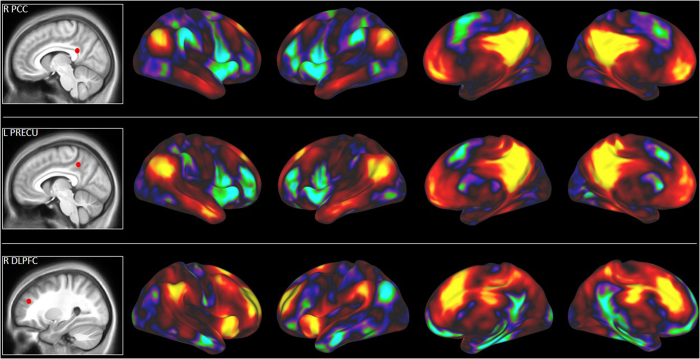
Resting-state functional connectivity (RSFC) maps with select default and executive network ROIs. Seeds were defined based on the whole-brain task contrast (alternate uses > object characteristics) and applied to an independent sample of participants (*n* = 42). Warm colors (red and yellow) reflect positive RSFC and cool colors (blue and green) reflect negative RSFC.

Our first set of analyses focused on the precuneus and PCC seeds. As expected, both seeds showed positive connectivity with other regions of the DMN, including MPFC, PCC, and bilateral IPL (see Fig. 2). The PCC and precuneus seeds also showed negative connectivity with regions of the ECN, including bilateral DLPFC and posterior parietal cortex; these regions also showed negative connectivity with salience network regions (bilateral insula and the ACC). Next, we examined resting-state connectivity with the right DLPFC seed. As expected, the right DLPFC showed positive connectivity with other ECN regions, including the left DLPFC and bilateral posterior parietal cortex, and negative connectivity with DMN regions, including MPFC, PCC, and bilateral IPL (see Fig. 2). The DLPFC seed also showed positive connectivity with regions of the salience network (bilateral insula and ACC), consistent with previous resting-state research^67^. The resting-state analysis thus confirmed the hypothesized resting-state network affiliations of the default and executive network ROIs.

### Seed-to-Voxel Analysis

**Figure 3:**
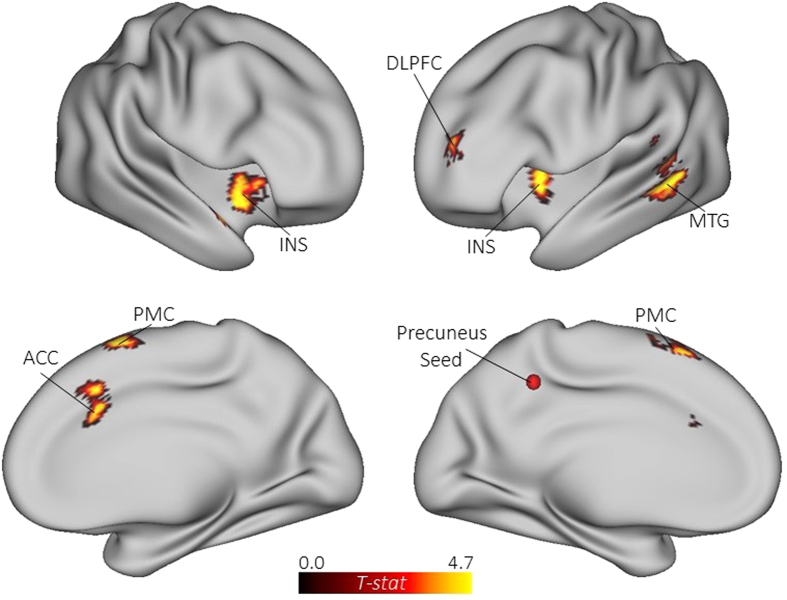
Functional connectivity maps for the general task contrast (alternate uses > object characteristics) with the left precuneus specified as a seed. ACC = anterior cingulate cortex; DLPFC = dorsolateral prefrontal cortex; INS = insula; MTG = middle temporal gyrus; PMC = premotor cortex.

**Figure 4:**
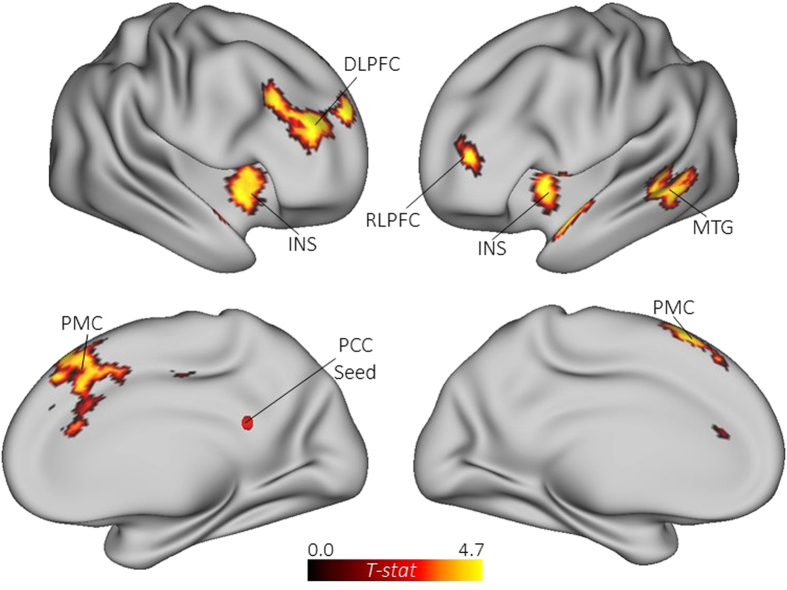
Functional connectivity maps for the general task contrast (alternate uses > object characteristics) with the right PCC specified as a seed. DLPFC = dorsolateral prefrontal cortex; INS = insula; MTG = middle temporal gyrus; PCC = posterior cingulate cortex; PMC = premotor cortex; RLPFC = rostrolateral prefrontal cortex.

Our next step was to analyze task-related connectivity associated with divergent thinking (alternate uses > object characteristics). The first seed-to-voxel analysis assessed connectivity between the precuneus seed and all other voxels (*p* < .05, FWE corrected). Results revealed increased functional connectivity between the precuneus and seven voxel clusters during divergent thinking, including regions within the executive network (right MFG; BA 9/10) and salience network (bilateral insula and ACC), as well as the left MTG and left pre-motor cortex (PMC; see Table 2 and Fig. 3). Next, we assessed connectivity between the right PCC and the rest of the brain. Similar to the precuneus seed, the PCC seed showed increased coupling with regions of the executive network (right DLPFC) and salience network (bilateral insula), as well as the left MTG and left PMC. Novel to this analysis, the PCC showed connectivity with a cluster of voxels in left rostrolateral prefrontal cortex (RLPFC; BA 10) and posterior parietal cortex (BA 31; see Table 2 and Fig. 4). These results extend the whole-brain MVPA by revealing direct functional connections between the core hubs of the DMN and ECN during divergent thinking.

**Figure 5:**
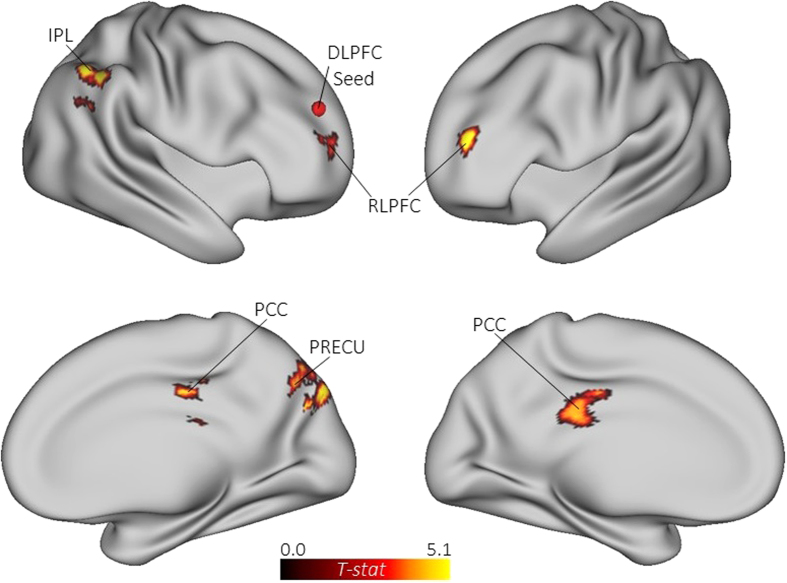
Functional connectivity maps for the general task contrast (alternate uses > object characteristics) with the right DLPFC specified as a seed. DLPFC = dorsolateral prefrontal cortex; IPL = inferior parietal lobe; PCC = posterior cingulate cortex; PRECU = precuneus; RLPFC = rostrolateral prefrontal cortex.

We then assessed task-related connectivity with the right DLPFC seed. In line with the above analyses, the right DLPFC showed increased connectivity with regions of the DMN, including the right IPL (BA 40), left PCC, and right precuneus (see Fig. 5); we also found connectivity between the right DLPFC seed and bilateral RLPFC (BA 10). Finally, we assessed connectivity with another region identified in the whole-brain analysis—left IFG (BA 45)—to compare task-related connectivity with results from a recent resting-state study showing increased coupling between this region and the DMN^68^. Results revealed increased connectivity between the left IFG and a cluster of voxels in the left IPL (BA 39), a core DMN region.

### ROI-to-ROI Temporal Connectivity

**Figure 6:**
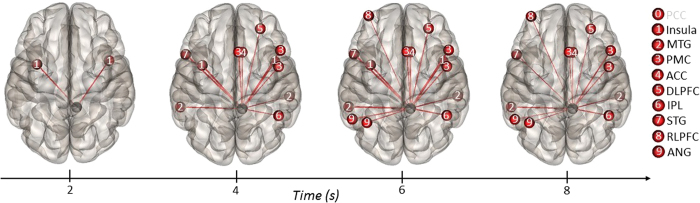
ROI-to-ROI temporal connectivity for the general task contrast (alternate uses > object characteristics) with the right PCC specified as the source ROI (black sphere) and all other ROIs specified as targets (red spheres). Regions labeled in black on the right show positive connectivity with the source ROI; regions labeled in gray were not significant.

We then assessed dynamic changes in functional connectivity across the duration of the task. All regions from the whole-brain MVPA were specified as ROIs (i.e., 10 mm spheres; see Table 1). The PCC, precuneus, and DLPPFC were specified as “source” ROIs, and the remaining ROIs were specified as “targets”. The first analysis explored temporal connections between the PCC source ROI and the other targets. During the first time window, the PCC showed increased functional connectivity with bilateral insula (see Fig. 6). The PCC remained connected to the bilateral insula during the second window, and showed further connectivity with the right DLPFC, ACC, and bilateral MTG, among other regions. This pattern of connectivity was sustained during the third window, with additional connectivity found with the left RLPFC and the left AG. The same pattern emerged during the next time window, with the exception that the PCC was no longer connected to bilateral insula; no significant connectivity differences were found during the final time window. The PCC thus showed early coupling with salience network regions (bilateral insula) and later coupling with an executive network region (right DLPFC).

**Figure 7:**
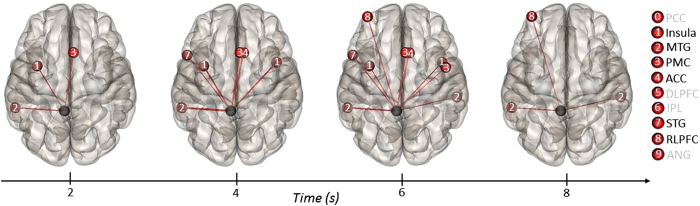
ROI-to-ROI temporal connectivity for the general task contrast (alternate uses > object characteristics) with the left precuneus specified as the source ROI (black sphere) and all other ROIs specified as targets (red spheres). Regions labeled in black on the right show positive connectivity with the source ROI; regions labeled in gray were not significant.

We then assessed temporal connections between the precuneus source ROI and the targets (see Fig. 7). During the first time window, the precuneus showed increased functional connectivity with the left insula, left MTG, and right PMC (see Fig. 7). During the second window, the precuneus showed sustained coupling with these regions and additional coupling with the right insula and left temporal pole (i.e., STG). This pattern persisted throughout the third time window, with additional connectivity found with the right MTG and left RLPFC. During the fifth time window, the precuneus showed connectivity with bilateral MTG and the left RLPFC; no significant connectivity differences were found during the final time window.

**Figure 8:**
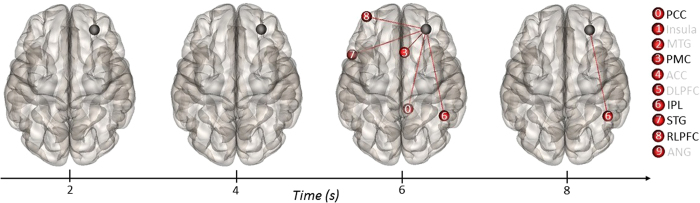
ROI-to-ROI temporal connectivity for general task contrast (alternate uses > object characteristics) with the right DLPFC specified as the source ROI (black sphere) and all other ROIs specified as targets (red spheres). Regions labeled in black on the right show positive connectivity with the source ROI; regions labeled in gray were not significant.

We then explored temporal connectivity with the right DLPFC target ROI. During the first two time windows (TRs 2-3), the RDLPFC did not show any significant connectivity differences with the target ROIs. However, during the third time window, the DLPFC showed increased connectivity with regions within the DMN, including the right PCC and right IPL, in addition to left RLPFC, left temporal pole, and right PMC (see Fig. 8). The DLPFC showed sustained coupling with the right IPL during the fourth time window, and no significant differences emerged during the final time window. Taken together, results from the temporal connectivity analyses revealed dynamic coupling between core regions of the default, salience, and executive networks at different stages of divergent thinking.

### Graph Theory Analyses

**Figure 9:**
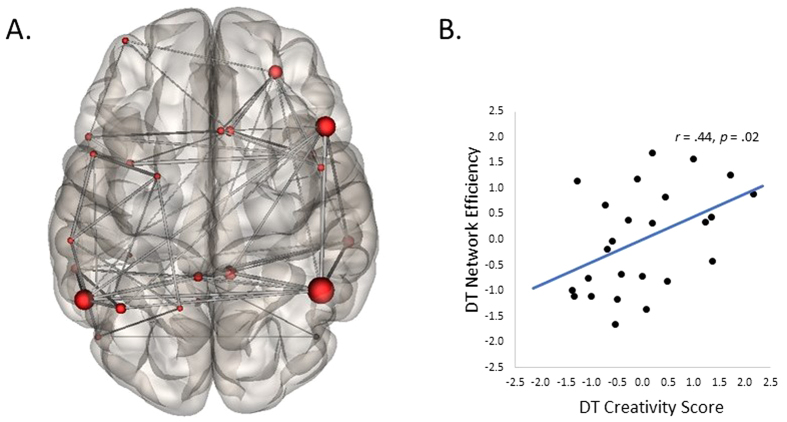
Graph theory analysis of the functional network associated with divergent thinking. (**A**) Nodes (ROIs from the whole-brain analysis) and edges (paths between the nodes) that were used to define the divergent thinking network. (**B**) Scatter plot depicting the correlation between composite creativity scores (i.e., average divergent thinking creativity ratings) and global efficiency of the divergent thinking network.

Finally, we computed global efficiency of the whole-brain network of ROIs (see Fig. 9A) and correlated global network efficiency with individual differences in divergent thinking ability (i.e., average creativity ratings to the alternate uses task completed outside of the scanner). As expected, global efficiency values were positively correlated with composite creativity scores (*r* = .44, *p* = .02)—as divergent thinking ability increased, the divergent thinking network showed greater efficiency (see Fig. 9B). This suggests that more creative participants exhibited more efficient information transfer across a network of brain regions linked to divergent thinking, including the core nodes of the default and executive networks.

## Discussion

The present study explored whole-brain functional connectivity associated with creative idea production. We identified a functional network related to divergent thinking, consisting of regions within the default (PCC, precuneus, and inferior parietal lobules) and executive (DLPFC) networks, among other regions. Resting-state functional connectivity analysis confirmed the underlying network affiliations of these regions, using data from an independent sample of participants. Seed-based analyses found increased connectivity between the DLPFC, PCC, and precuneus, and temporal analyses revealed dynamic coupling between these regions at different stages of the divergent thinking task. The results extend past research by revealing functional connections between regions commonly associated with creative cognition. Moreover, functional connectivity between hubs of large-scale networks points to a greater cooperation between networks associated with cognitive control and spontaneous thought processes.

The whole-brain MVPA revealed a distributed network associated with divergent thinking (see Fig. 1). This network consisted of several core default regions, including the precuneus, PCC, and bilateral IPL, as well as the right DLPFC, a core region of the executive network^67^. The network also included the hubs of the salience network (bilateral insula and ACC), as well as several other temporal regions (e.g., bilateral MTG). Activation of temporal regions is consistent with previous neuroimaging studies of creative cognition^36^, and may reflect increased demands on memory retrieval mechanisms common to the temporal lobes. Together, results from the whole-brain analysis indicates a greater cooperation between brain regions involved in spontaneous thought, cognitive control, and semantic memory retrieval. These findings are consistent with the emerging literature on the cooperative role of default and executive networks during cognitive states that involve focused internal attention^8^.

Seed-based and ROI-to-ROI analyses were conducted to explore further connections between specific default and executive regions identified in the whole-brain analysis. We found increased connectivity between the right DLPFC seed and regions of the default network, including the right IPL and the precuneus, as well as connectivity between the PCC seed and the DLPFC. These results extended the whole-brain analysis by showing direct connections between default and executive network regions during the task. Such findings provide support for the notion that creative thought involves cooperation between spontaneous and controlled processes^17^,^18^,^42^,^69^.

We also found increased connectivity between default regions (PCC and precuneus) and regions of the salience network (dorsal ACC and bilateral insula). The salience network is involved in reallocating attentional resources to salient environmental events^2^, and it is thought to play a central role in dynamic switching between other brain networks, especially the DMN and the ECN^2^,^8^. In this context, functional connectivity between default, salience, and executive regions may reflect dynamic switching between large-scale networks during divergent thinking.

Further support for this notion comes from results of the ROI-to-ROI temporal connectivity analyses. Here, we examined changes in connectivity between default and executive network ROIs across the duration of the task. We found that the PCC was more strongly connected to salience network regions (i.e., bilateral insula) at the beginning of the task, followed by stronger connections with executive network regions (i.e., right DLPFC). Early coupling of the PCC with the salience network may provide an intermediate mechanism that facilitates later coupling with the executive network. Moreover, we found differential coupling between the right DLPFC seed and default network regions (i.e., PCC and right IPL). Interestingly, the DLPFC only showed connectivity with default regions during the second half of the task (i.e., TRs 4-5), pointing to a potential role executive processes at later stages of divergent thinking. Taken together, such dynamic coupling may reflect cooperation between brain networks associated with cognitive control and spontaneous thought, consistent with recent theorizing on the role of attention in creative cognition^14^,^16^,^17^,^18^.

The present results raise the question of how such typically opposing networks cooperate in the brain. A large body of resting-state and task-based research has reported an antagonistic relationship between the DMN and ECN. During working memory performance, for example, the ECN shows increased activation while the DMN deactivates^2^, presumably indicating the suppression of task-unrelated thought during cognitive control^67^. At the same time, a growing literature points to certain conditions that foster a greater cooperation between these typically opposing networks (e.g.,^54^). Such findings suggest that DMN and ECN regions show increased coupling when attention is focused on internally-directed processes^8^.

Like other types of self-generated thought (e.g., future thinking), creative thinking may require focused internal attention (cf. 70, 71). But the need for additional executive control may differentiate creative cognition from other modes of self-generated thought. For example, during a divergent thinking task, people typically begin by retrieving known uses for a given object (e.g., a brick, “build a house”) before eventually shifting to more elaborate and effective semantic search strategies^42^,^47^. Executive control can mitigate these early sources of interference by suppressing salient conceptual knowledge (e.g., typical uses for an object; 43, 48) and facilitating flexible switching between semantic categories during memory retrieval^48^. Co-activation of default and executive networks may thus reflect both focused internal attention and the executive control of thought content.

The present study extends recent research on resting-state functional connectivity and divergent thinking ability^72^. Beaty and colleagues contrasted intrinsic connectivity networks of high- and low-divergent thinking ability groups and found that high divergent thinking ability was related to greater connectivity between the inferior frontal gyrus and the default network. The IFG is involved in several executive processes, such as controlled memory retrieval^68^ and pre-potent response inhibition^73^. Hence, connectivity between the IFG and the DMN was interpreted as a greater ability of highly creative individuals to exert top-down control over imaginative processes stemming from the DMN. A similar pattern was observed in the present study: we found increased connectivity between the left IFG and the left IPL. It’s worth noting, however, that the IFG region found in the present study was located in BA 45 (pars triangularis), whereas the IFG region used in the resting-state study was in BA 47 (pars orbitalis). Nevertheless, the results of both studies suggest that creative thought may rely on functional coupling of brain regions associated with cognitive control and spontaneous thought.

## Limitations and Future Directions

The present study explored dynamic interactions between brain regions during performance on a creative thinking task. We found increased functional connectivity between regions of the default and executive networks, pointing to cooperation between large-scale networks underlying creative idea production. One notable limitation of the study was our inability to capture participant responses in the scanner, which would have shed light on task compliance and further permitted parametric analyses of brain activity related to the creative quality of responses. Yet the high correlation between individual differences in divergent thinking ability, assessed outside the scanner, and global efficiency of the divergent thinking network suggests that participants were indeed engaged in divergent thinking during the task, and that the integrity of the network was sensitive to the creative ability of participants.

The study was also limited to the use of a single assessment (an alternate-uses divergent thinking task) to indicate a rather broadly defined construct (creativity). Future research should examine functional connections among brain regions during other creative thinking tasks and in relation to creative performance in specific domains, such as musical improvisation^74^. This approach would shed light on whether connectivity between default and executive networks is exclusive to divergent thinking, or whether such connectivity reflects a domain–general network underlying a range of creative thought processes. In addition, future research should attempt to clarify whether creative cognition differs from other imaginative processes (e.g., future thinking) in terms of executive involvement. We assumed that divergent thinking requires greater executive activity to manage internal sources of interference, but it remains to be seen whether such processes are more relevant for creative thought compared to other self–generated thought processes.

## Additional Information

**How to cite this article**: Beaty, R. E. *et al*. Default and Executive Network Coupling Supports Creative Idea Production. *Sci. Rep*. **5**, 10964; doi: 10.1038/srep10964 (2015).

## Figures and Tables

**Table 1:** Peak activation clusters resulting from the whole-brain MVPA task contrast (alternate uses > object characteristics). Lobes are shown in italics

	Left Hemisphere					Right Hemisphere				
Lobe/Region	BA	x	y	z	voxels	BA	x	y	z	voxels
*Frontal*										
DLPFC						9	28	44	26	599
IFG	45	−50	34	6	101	–	–	–	–	–
ACC	–	–	–	–	–	32	8	18	44	58
RLPFC	10	−38	58	18	318					
MFG	6	−24	−2	54	677	6	4	18	64	104
	6	−52	8	22	324	6	48	2	42	115
	–	–	–	–	–	8	50	20	38	63
*Parietal*										
PRECU	31	−6	−46	44	127	–	–	–	–	–
PCC	–	–	–	–	–	31	8	−44	24	2605
IPL	40	−62	−30	38	362	40	48	−52	44	1836
ANG	39	−40	−60	52	324	–	–	–	–	–
	39	−56	−56	34	63	–	–	–	–	–
RSC	30	−6	−56	10	368					
SPL	7	−14	−60	62	49	–	–	–	–	–
*Temporal*										
INS	13	−36	4	−4	87	13	44	8	−2	743
MTG	21	−60	−42	−2	452	21	60	−30	−14	220
ITG	37	−36	−36	−14	62	–	–	–	–	–
STG	38	−54	16	−8	168					
*Occipital*						–	–	–	–	–
MOG	19	−50	−72	0	691	19	46	−72	12	67

ACC = anterior cingulate cortex; ANG = angular gyrus; DLPFC = dorsolateral prefrontal cortex; IFG = inferior frontal gyrus; INS = insula; IPL = inferior parietal lobule; ITG = inferior temporal gyrus; MFG = middle frontal gyrus; MOG = middle occipital gyrus; MTG = middle temporal gyrus; PCC = posterior cingulate cortex; PRECU = precuneus; RLPFC = rostrolateral prefrontal cortex; RSC = retrosplenial cortex; SPL = superior parietal lobe; STG = superior temporal gyrus.

**Table 2:** Seed-to-voxel results with the precuneus and PCC specified as seeds (shown in italics).

Seed/Lobe/Region	BA	x	y	z	voxels
1. L PRECU					
*Frontal*					
L DLPFC	9/10	−30	52	26	185
ACC	32	8	18	34	156
L PMC	6	−2	12	74	329
*Temporal*					
L INS	13	−36	4	2	149
R INS	13	42	8	−6	352
L MTG	21	−58	−40	2	347
2. R PCC					
*Frontal*					
R DLPFC	9	36	44	20	796
L RLPFC	10	−36	36	14	114
R PMC	6	2	38	42	984
*Temporal*					
L INS	13	−46	10	−2	679
R INS	13	42	6	2	404
L MTG	21	−56	32	−2	487
*Parietal*					
R PCC	31	30	−22	36	127
3. R DLPFC					
*Frontal*					
R RLPFC	10	34	54	28	127
L RLPFC	10	−34	54	22	226
*Parietal*					
R PRECU	7	10	−78	40	432
L PCC	23	−6	−30	26	159
R IPL	40	56	−46	48	375

ACC = anterior cingulate cortex; DLPFC = dorsolateral prefrontal cortex; INS = insula; IPL = inferior parietal lobule; MTG = middle temporal gyrus; PCC = posterior cingulate cortex; PMC = premotor cortex; PRECU = precuneus; RLPFC = rostrolateral prefrontal cortex.
